# Investigation of Different Iontophoretic Currents Profiles for Short-Term Applications in Cosmetics

**DOI:** 10.3390/pharmaceutics10040266

**Published:** 2018-12-07

**Authors:** Jennyfer Cázares-Delgadillo, Lien Planard-Luong, Sébastian Gregoire, César E. Serna-Jiménez, Mayank Singhal, Yogeshvar N. Kalia, Virginia Merino, Matilde Merino-Sanjuán, Amparo Nácher, Ma. Amparo Martínez-Gómez, Veronique Burnier-Yalaoui, Philippe Barbarat

**Affiliations:** 1 Instrumental Cosmetics, Applied Research, L’Oréal, 94550 Chevilly-Larue, France; lien.planard-luong@rd.loreal.com (L.P.-L.); veronique.burnier@rd.loreal.com (V.B.-Y.); philippe.barbarat@rd.loreal.com (P.B.); 2 Advanced Research, L’Oréal, 93600 Aulnay-sous-Bois, France; sebastien.gregoire@rd.loreal.com; 3 School of Pharmaceutical Sciences, University of Geneva & University of Lausanne, 1205 Geneva, Switzerland; Cesar.Serna@hotmail.es (C.E.S.-J.); mayanksinghal87@msn.com (M.S.); Yogi.Kalia@unige.ch (Y.N.K.); 4 Instituto Interuniversitario de Investigación de Reconocimiento Molecular y Desarrollo Tecnológico (IDM), Universitat Politècnica de València, Universitat de València, 46100 Valencia, Spain; Virginia.Merino@uv.es (V.M.); Matilde.Merino@uv.es (M.M.-S.); amparo.nacher@uv.es (A.N.); 5 Departament de Farmàcia i Tecnologia Farmacèutica i Parasitologia, Universitat de València, 46100 Valencia, Spain; 6 Servicio de Farmacia: Hospital Universitario Doctor Peset, 46017 Valencia, Spain; martinez_margoma@gva.es

**Keywords:** iontophoresis, topical penetration, ascorbic acid, ellagic acid, pulsed current, galvanic current, square unipolar pulse current

## Abstract

This study aimed at investigating the effect of electrical current profile upon the iontophoretic transport of (i) ascorbic acid (AA) and (ii) ellagic acid (EA), into porcine skin in vitro, and the impact of the physicochemical properties of both actives on their mechanism of transport when formulated in cosmetic compositions. The experiments were performed using a proprietary iontophoretic device containing a roller to apply the formulation. Three current profiles were tested: (i) galvanic direct current (DC), (ii) square unipolar pulse current (SPC), and (iii) galvanic direct current (DC) + pulse current (PC). The skin samples were collected at different sampling points, extracted and analyzed by HPLC. Results suggested that the DC + PC mode for only 5 min was able to significantly increase the delivery of AA from o/w cosmetic compositions. The use of this current profile might improve the skin penetration of AA due to electromigration and passive diffusion, the latter being facilitated by the physical enhancement method. The SPC mode significantly improved the passage of EA in its neutral form from cosmetic o/w formulations by electroosmosis. Tailoring specific electrical current modes considering the ionization state of active ingredients would allow the design of short and personalized cosmetic treatments that significantly improve the penetration efficiency of the active ingredients and possibly reduce the doses applied.

## 1. Introduction

**Figure 1:**
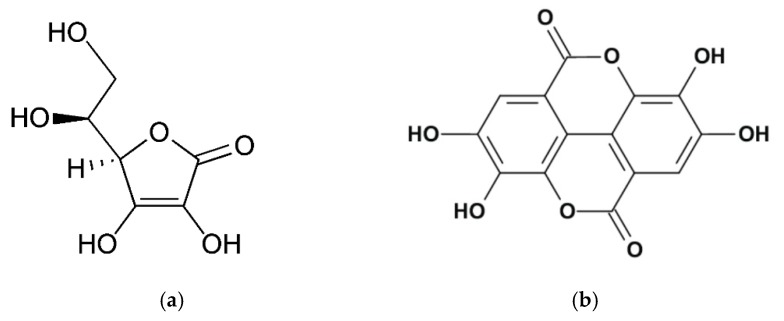
(**a**) Ascorbic acid (AA) chemical structure; (**b**) Ellagic acid (EA) chemical structure.

l-Ascorbic acid (AA; Vitamin C) and ellagic acid (EA) are used to decrease age-related skin problems that may negatively impact skin appearance, see Figure 1. Ascorbic acid and its various derivatives behave as antioxidants against oxidative stress in humans and may also act as free radical scavengers. In addition, AA plays an important role in the synthesis of collagen and it is known to prevent skin darkening and reduce oxidized melanin. One problem, however, is that AA is easily oxidized and, therefore, loses function.

EA (2,3,7,8-tetrahydroxy-chromeno[5,4,3-cde]chromene-5,10-dione) is a phenolic compound that enhances the levels of antioxidant enzymes and protects against UVB. Moreover, it has an anti-inflammatory action and wound healing effects. However, it is poorly absorbed and eliminated from the body with a half-life of 8.4 h [1]. Furthermore, it is poorly soluble in aqueous media and it is usually formulated below pH 6 to avoid coloring and precipitation in cosmetic formulations.

Iontophoresis is a technique that allows the movement of charged or neutral species across the skin by means of a small electric potential using two principal mechanisms: electromigration and electroosmosis [2]. Upon the application of the electrical potential, cations electromigrate from the anode to the skin, and similarly, anions move away from the cathode. Uncharged molecules are transported by electroosmosis in the anode-to-cathode direction by the convective solvent flow generated at physiological pH [3].

A few studies have reported on the delivery of ascorbic acid using galvanic direct current (DC) profiles to achieve a clinical improvement in melasma [4,5,6,7]. An increase in skin collagen synthesis induced by an AA derivative after “frequent-reversal bipolar electrical iontophoresis” has also been reported [8]. The penetration of EA has been studied in vitro from ointment containing polyethylene glycols (PEGs) and pomegranate rind extract for the treatment of dermal diseases [9], and from niosomal formulations from the mixture of Span 60 and Tween 60 [10], to increase dermal delivery of the active ingredient. EA has not been tested so far in combination with non-invasive active technologies.

AA is a negatively charged molecule at physiological pH (MW: 176.12; pKa1: 4.17 pKa2: 11.57) and can be delivered to the skin by electromigration using cathodal iontophoresis. EA has a phenolic group (MW: 312.197; pKa1 = 6.69; pKa2 = 7.45; pKa3 = 9.61; pKa4 = 11.50), which is negatively charged at physiological conditions and practically neutral bellow pH 5.0, and, hence, is also a good candidate for cathodal iontophoretic transport from aqueous media.

Here, the use of a dynamic current application was investigated by using a roll-on applicator for optimized treatment according to the programs and skin areas to being treated. An important point was to investigate how the use of complex formulations containing cosmetic excipients, affected the electrotransport behavior. The amount of product delivered was controlled by an electronic system. The current generator in the device provided (i) galvanic constant DC (+ or −) mode, programmed for an iontophoresis mode (ii) square unipolar (+ or −) pulse current (SPC)—unipolar iontophoresis—, and (iii) DC coupled with pulsed current (PC) (+ or −) for an ionto-stimulation mode. The duration and frequency of these pulses were programmable and enabled customized delivery of the active cosmetic ingredients to the skin.

In summary, this paper describes the effect of the short-duration application of different current profiles DC, SPC, and DC + PC using a dynamic applicator (roll-on type) on the skin penetration of AA and EA, and the impact of the physicochemical properties of both actives when formulated in cosmetic compositions on their electrotransport.

## 2. Materials and Methods

### 2.1. Chemicals

Pure AA (99.9%) (DSM Nutritional Products France, Courbevoie, France), pure EA (98.9%) (Lion Corporation, Tokyo, Japan), AA Formula 1 (AA 5%; o/w emulsion; pH 6.7 ± 0.3), AA Formula 2 (AA 10%; o/w emulsion; pH 6.0 ± 0.3), EA solution (0.2% in HEPES 20 mM/propylene glycol (26/74) at pH 4.5 or pH 7.0), EA Formula 1 (0.5% EA; o/w emulsion; pH 4.7 ± 0.3), EA Formula 2 (0.5% EA; o/w emulsion; pH 4.8 ± 0.3), were provided by L’Oréal (Chevilly-Larue, France). Monobasic potassium phosphate was purchased from Acros Organics (Geel, Belgium) and sodium metabisulfite was obtained from Sigma Aldrich (Steinheim, Germany). All solutions were prepared using deionized water (Millipore Milli-Q Gard 1 Purification Pack resistivity >18 MΩ.cm; Zug, Switzerland). All other chemicals were at least of analytical grade.

### 2.2. Profiles of the Electric Currents

**Figure 2:**
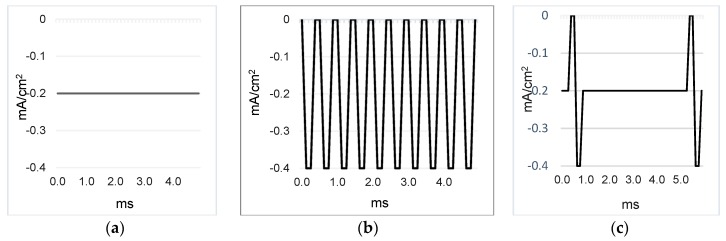
Negative polarity current profiles: (**a**) galvanic direct current (DC); (**b**) square unipolar pulse current (SPC); (**c**) galvanic direct current and pulse current (DC + PC).

The three current profiles used were galvanic direct constant current (DC) at 0.2 mA/cm^2^ or 0.3 mA/cm^2^; square unipolar pulse current (SPC) (2000 Hz, duty cycle 50%), and galvanic direct current (DC) at 0.2 mA/cm^2^ + pulse current (PC) 200 Hz, pulse wide 500 µs. All PC profiles were programmed with average pulse values at 0.2 mA/cm^2^. The polarity was either positive (anodal iontophoresis) or negative (cathodal iontophoresis), see Figure 2, depending on the conditions used.

### 2.3. Skin Preparation

Porcine ears were obtained from a local slaughter (CARRE, Rolle, Switzerland) and the Faculty of Medicine (University of Valencia, Spain) within a few hours of sacrifice and were stored at 4 °C until processing.

The whole skin was removed carefully from the outer region of the porcine ear or porcine flap, and separated from the underlying cartilage either with a scalpel or with a dermatome (dermatome at Aesculap-Wagner C. GA 176, B. Braun Surgical S.A., Barcelona, Spain), respectively. The skin pieces were stored from −20 to −25 °C until use.

### 2.4. Analytical Method

The methods employed for analysis of AA and EA are detailed in Table 1.

### 2.5. Skin Extraction Procedure (n = 3)

AA was extracted from the skin using the extraction medium: acetonitrile: KH_2_PO_4_ 0.05 M and Na_2_S_2_O_5_ 0.5% (75:25). The skin extraction recovery was determined by spiking the skin with 50 µL of 1 mg/mL stock solution. When it was dry, the skin was cut into small pieces and kept in contact under stirring with the extraction medium for either 1 or 4 h. Although the extraction efficiency can sometimes be increased with longer incubation times, the risk of an increased degradation of the studied active ingredient may occur. After extraction, the samples were filtered through a nylon filter membrane with a pore size of 0.22 μm.

EA was extracted by methanol as a solvent. The skin recovery was determined by spiking the skin with 20 µL of the solvent. The skin samples were kept in contact with the extraction medium for 24 h. After extraction, the samples were filtered through a polyvinylidene difluoride (PVDF) membrane filter of 0.45 µm and analyzed.

All samples were analyzed by the validated HPLC-UV methods.

### 2.6. Skin Delivery Studies (n = 6)

These aimed at quantifying the electrically-assisted skin penetration of AA and EA from cosmetic formulations at a current density of 0.2 mA/cm^2^ with formulation application for 5 min using three negative current application profiles, see Table 2. Passive penetration using the same set-up—with lateral movement—but in the absence of current application was used as the control. There were three control experiments, one for each formulation and one to determine the endogenous AA in the skin.

EA Formula 1 was subjected to similar conditions to those described above but using positive SPC and DC + PC application profiles. Passive diffusion (one experiment) was used as the control, see Table 2.

Before the experiments, each of the full-thickness skin samples were visually checked for their integrity. Each piece of porcine ear skin (approximately 2 × 4 cm) was equilibrated by soaking in PBS for 30 min at room temperature. After the equilibration period, the samples were fixed onto a polystyrene support. The polystyrene had a small wedge of material removed and the space created contained a small piece of aluminum foil with the skin on top. The aluminum foil piece was connected to the proprietary device by means of a cable with crocodile clips in both ends to complete the electrical circuit. The cosmetic formulations were applied to an exposed skin surface of 0.8 cm × 2.4 cm (1.92 cm^2^), delimited by means of an adhesive tape (3M 9773). A total of 100 mg of AA Formula 1 or AA Formula 2 were applied to the exposed skin surface. In addition, the roll-on was filled with the formulation after each replication in order to ensure good contact with the formulation present on the skin surface. The set-up was placed on a metallic support stand in a mechanical shaker which made small forward and backward movements (60 times/min and 2.4 cm in each direction) during the entire application period. Hence, the roller was kept in motion and the pre-programmed number of horizontal backwards and forwards movements ensured coverage of the entire skin surface and reproducibility of the movements between the samples and the studies.

In the case of EA, the skin samples had a slightly smaller area (approximately 1 × 5 cm) and were equilibrated using a tissue soaked with saline solution (NaCl 0.9%) at 32 °C for 1 h. The cosmetic formulations were applied to the exposed skin surface of 0.5 cm × 4 cm (2 cm^2^), delimited by means of a Teflon piece. A total of 100 mg of EA Formula 1 was applied to the exposed skin surface. The remaining conditions were similar to those described above. During both studies, the current intensity was monitored.

The effect of pH on the iontophoretic skin transport of EA formulated in simplex solutions and an o/w cosmetic formulation (EA Formula 2) was also investigated. Dermatomed porcine flap slices of 600 µm thickness were placed between the donor and receptor compartments of the Franz diffusion cell (0.789 cm^2^) and both compartments were filled with 1 and 6 mL of buffer (NaCl 150 mM/HEPES 20 mM pH 7.4), respectively. Skin resistance (≥4 kΩ) and skin temperature (32 ± 1 °C) were measured. Afterwards, the donor compartment was emptied and refilled with 1 mL of either EA solutions containing 0.2% EA in HEPES 20 mM/propylene glycol (26/74) at pH 4.5 or pH 7.0, or EA Formula 2 (0.5% EA; o/w emulsion; pH 4.8 ± 0.3), see Table 2. Salt bridges (SB) were used in the set-up to separate the electrical and donor compartments and eliminate competition from Cl^−^ ions. Positive and negative DC current profiles were applied at 0.3 mA/cm^2^ for 10 min or 20 min via Ag/AgCl electrodes connected to a power supply (Kepco BHK-MG 0-2000V, New York, NY, USA), see Table 3. Passive penetration using the same set-up but in the absence of current application was used as the control.

Upon completion of all the experiments, the formulations were removed from the skin surface with a soft tissue paper which was then cleaned with running water to remove any residue.

In the case of AA, the skin samples were cut into small pieces and placed for 1 h in an extraction medium (acetonitrile:KH_2_PO_4_ 0.05 M and Na_2_S_2_O_5_ 0.5% (75:25)) to extract the AA deposited within the membrane. The extraction medium was filtered using a nylon filter membrane with a pore size of 0.22 μm. Skin extraction was also performed using untreated skins to confirm endogenous levels of ascorbic acid present in the skin (*n* = 3). In all cases, skin deposition samples were analyzed immediately after the extraction.

With respect to EA, the skin samples were cut and placed in the extraction medium (methanol) for 24 h to obtain a higher amount. The extraction medium was filtered through a PVDF membrane with a pore size of 0.45 µm and the samples were analyzed.

### 2.7. Stability of l-Ascorbic Acid (AA) and Ellagic Acid (EA) in the Presence of Electric Current (n = 3)

Stability assays were performed to measure any change in the concentration of AA and EA in each formulation following current application at 0.2 mA/cm^2^ for 5 min. The stability was tested using each current profile DC, SPC, and DC + PC. Accordingly, aliquots of AA or EA solutions were was sampled from the applicator and diluted appropriately to be analyzed by HPLC. In addition, EA stability in the presence of current using a DC profile was evaluated to be 0.3 mA/cm^2^ using Ag/AgCl electrodes connected to a power supply (Kepco BHK-MG 0-2000V) for 30 min. At the end of the assay, the samples were kept at 4 °C until analysis.

### 2.8. Statistics

Results were plotted as skin deposition (expressed as percentage of the amount applied to the skin) or deposited amount (expressed in µg/cm^2^). All values have been presented as mean ± standard deviation (SD). A student’s *t*-test and an analysis of variance (ANOVA) test were performed to investigate significant difference between the different experiments. A statistically significant difference was shown by *p* values < 0.05.

## 3. Results

### 3.1. AA Assessment

#### 3.1.1. Analytical Method

Intra-day validation was performed by analyzing five standards from 10 to 100 μg/mL (*n* = 5) prepared in acetonitrile: KH_2_PO_4_ 0.05 M and Na_2_S_2_O_5_ 0.5% (75:25). The analytical method showed good linearity (*r*^2^ = 0.998) and the variation in the accuracy and precision was less than 10%.

#### 3.1.2. Stability of AA in the Presence of Current

**Figure 3:**
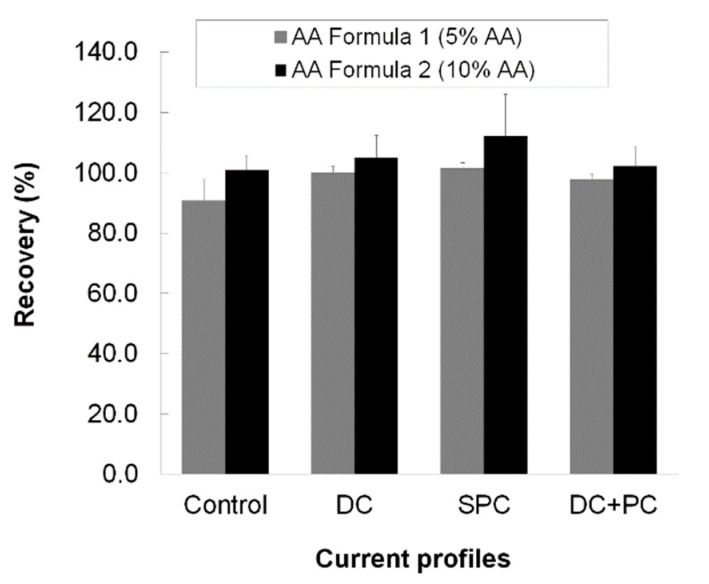
Stability of AA in the presence of the different current profiles.

The stability was expressed as the recovery of ascorbic acid with respect to the initial concentration in the formulation. The results obtained showed that ascorbic acid was stable in the presence of an applied electric current, as evidenced by a 100% recovery for all the current profiles, see Figure 3. No statistically significant differences were found in the stabilities following application of the different current profiles (ANOVA; *p* < 0.05).

#### 3.1.3. Skin Delivery

Due to the sensitivity of ascorbic acid to oxidation, sodium metabisulfite [11] was added to the extraction solvent-acetonitrile: KH_2_PO_4_ 0.05 M and Na_2_S_2_O_5_ 0.5% (75/25). All dilutions were performed with the same solution. The skin extraction recovery (as the % of the initial amount) was 84.9 ± 5.6% and 73.6 ± 13.5% for 1 and 4 h, respectively (*n* = 3). Given the recovery obtained with extraction for 1 h and the risk of degradation of AA, it was decided that an incubation period of 1 h should be used to extract it from the skin samples.

**Figure 4:**
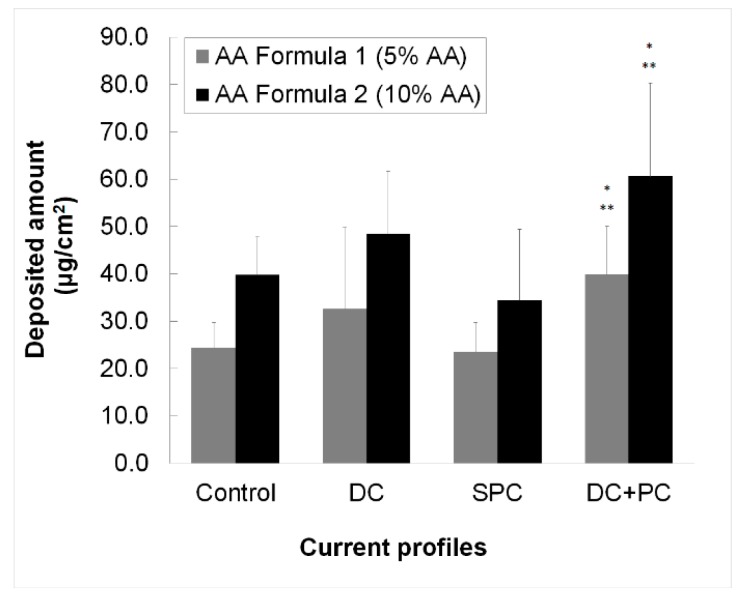
Deposited amount of AA obtained with AA Formula 1 (5%) and AA Formula 2 (10%) at each current profile (0.2 mA/cm^2^) for 5 min iontophoresis (*n* = 6). * Denotes statistically significant difference with respect to the corresponding control; ** denotes statistically significant difference with respect to the SPC current mode applied to the corresponding formula.

The quantification of the endogenous AA showed that 6.8 ± 1.4 μg/cm^2^ (*n* = 3) was present into the skin. Absolute skin deposition of AA (i.e., without subtraction of the mean endogenous amounts) obtained for the formulations using each current profile is shown in Figure 4. With both AA Formula 1 and AA Formula 2, there were no statistically significant differences following comparison using ANOVA (*p* < 0.05). However, a direct comparison using a Student’s *t*-test showed that there was a statistically significant difference between DC + PC and the control and between DC + PC and SPC for both Formulas (*p* < 0.05). The data shown in Figure 4 was calculated using an extraction volume of 4 mL.

### 3.2. EA Assessment

#### 3.2.1. Analytical Method

Intra-day validation was performed by analyzing 10 standards from 0.05 to 370 μg/mL (*n* = 3) prepared in HEPES 20 mM /propylene glycol (26/74). The analytical method showed good linearity (*r*^2^ = 0.99) and the variation in the accuracy and precision was less than 6%.

#### 3.2.2. Stability in the Presence of Current

EA was stable in the presence of an electric current at 0.03 mA/cm^2^ and 0.3 mA/cm^2^ during 30 min period of the application tested, obtaining a fraction of the initial EA concentration of 100.06 ± 0.38 (0.38%) and 99.79 ± 0.36 (0.36%), respectively. (*p* < 0.05; ANOVA).

#### 3.2.3. Skin Delivery

The skin extraction recovery from skin samples was 35.98 ± 3.23 (9%). This percentage of recovery was used to correct data from the permeability assays.

**Figure 5:**
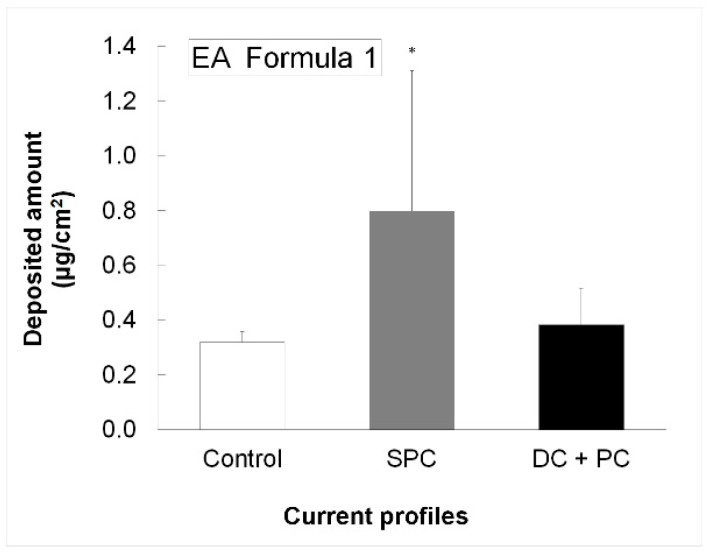
Deposited amount of EA obtained with EA Formula 1 at 0.2 mA/cm^2^ applied for 5 min (*n* = 6). * Denotes statistically significant difference with respect to the corresponding control.

The passage of EA was evaluated to determine the best penetration enhancement conditions, from a cosmetic composition using a short application time and different current profiles. Analysis of the skin profiles suggested that the SPC profile applied during 5 min at 0.2 mA/cm^2^ improved the topical transport of EA compared to that obtained from its passive application. The combined positive DC + PC profile did not show any particular advantage in increasing the penetration of the phenolic compound (*p* < 0.05; ANOVA), see Figure 5.

The iontophoretic delivery efficiency using the DC profile for EA delivery from simplex solutions was also investigated in order to understand the behavior of the molecule when present in its different ionized states. EA concentration was fixed at 0.2% due to its poor solubility in the media (water/propylene glycol 26/74). An o/w cosmetic formulation (EA Formula 2) was also evaluated in order to observe the passage of the molecule from an o/w cosmetic formulation at the selected current profile (DC) and to see its influence with respect to the other current application profiles. In this case, longer application times (10 min and 20 min) and higher current density (0.3 mA/cm^2^) were selected in order to ensure the detection and quantification of the molecule.

**Figure 6:**
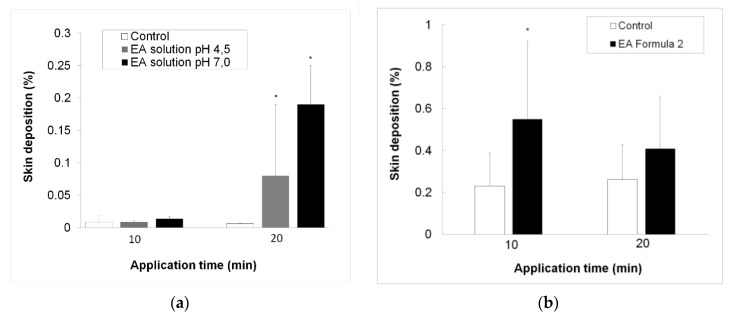
(**a**) Comparison of the deposition of 0.2% EA solution at pH 4.0 and pH 7.0 at 0.3 mA/cm^2^ applied for 10 min and 20 min iontophoresis; (**b**) comparison of the deposition of 0.5% EA Formula 2 at pH 4.8 at 0.3 mA/cm^2^ applied for 10 min and 20 min. The amounts of EA deposited are expressed as percentages of the amounts applied to the skin (*n* = 6). * Denotes statistically significant difference with respect to the control at the corresponding application time.

The results showed that a positive DC profile promoted the skin accumulation of EA. This effect was observed when the molecule was either applied in solution or administered as an o/w formulation (EA Formula 2). The skin depositions obtained in the different assays were compared by means of a Student’s *t*-test for non-paired data. The results showed statistically significant improvements in penetration for both charged (pH 7.0) and uncharged EA (pH 4.5) compared to the control; the effect was more marked after 20 min current application, see Table 4. Increasing the solution’s pH from 4.5 to 7 significantly increased the effect (*p* < 0.05; ANOVA), as shown in Figure 6a.

Figure 6b compares the deposition of 0.5% EA Formula 2 after iontophoresis at 0.3 mA/cm^2^ at two timings (10 min and 20 min). Statistical differences were obtained only between passive diffusion and iontophoresis for 10 min. The amount of EA accumulated in the skin is higher than that observed when applied in solution due to the dose applied with EA Formula 2 (2.5-times higher than that of the solution) (*p* < 0.05; ANOVA).

## 4. Discussion

Ascorbic acid appears as a good model candidate for iontophoretic delivery. It possesses good aqueous solubility, is ionizable and carries a negative charge in aqueous systems at physiologically acceptable pH, and can be formulated at high concentrations. However, from the different current profiles and conditions used here, only DC + PC showed some superiority over the control. The other two current profiles (DC and SPC) were statistically equivalent to control for both formulations. Therefore, it is curious as to why more significant improvements were not observed upon constant current application using these conditions. Indeed, it has been shown in previous internal studies that cathodal iontophoresis of ascorbyl phosphate from aqueous solutions is significantly improved by iontophoresis. AA Formula 1 and AA Formula 2 have complex compositions with many different excipients and were developed to facilitate the passive administration of ascorbic acid into the skin. Now, if the ascorbic acid is partially ionized, it cannot completely benefit from electromigration from the cathode upon application of a constant electric current. In this case, given that ascorbic acid was partially uncharged in these formulations, which were iontophoresed at the cathode, it is possible that the results observed with DC + PC were not solely due to electromigration but might be attributed to the effect of this current profile in increasing the passive permeability of the skin. DC + PC might function as a physical enhancement method to facilitate passive diffusion through a modified skin barrier as is the case for electroporation. Thus, it is probably premature to state that DC + PC is the most appropriate method to be used in order to improve the iontophoretic delivery of ascorbic acid (or its derivatives) from any type of formulation. The combination of galvanic and pulsatile current profiles may be more convenient for cosmetic applications as those may avoid skin irritation (usually perceived when using a galvanic direct current). However, the ionization state of the molecule (i.e., fraction ionized), in particular, of a potentially negatively charged species, in the formulation must be known in order to determine whether it will benefit from iontophoresis and, importantly, to select the polarity to be used for delivery. A cosmetic formulation that contains fully ionized, negatively charged AA species is being studied in order to maximize the benefits of the technique and to reach a better understanding of the effect of combined iontophoresis profiles on ascorbic acid cosmetic treatments.

Due to the physicochemical properties of EA, particularly the stability issues in acid media, the influence of different current profiles was evaluated on the active penetration from an o/w cosmetic composition. The results showed that positive SPC applied for a very short while (5 min) improved the topical transport of EA compared to its passive application. EA is in its uncharged form and was probably enhanced by electroosmosis but also by passive diffusion due to the physical effect of this current profile.

When EA was delivered from simplex solutions, it was practically unionized at pH 4.5, whereas, at pH 7, the molecule is predominantly negatively charged. The analysis of the skin penetration profiles suggested that the ionized form of EA was significantly transported when applied from the negative electrode, compared to its neutral form delivered from the positive electrode. However, the amount penetrated is small. The passage of the active ingredient at pH 4.5 from the negative electrode was not evaluated. It was assumed that at such pH, electroosmosis from the negative electrode would be similar to that from the positive electrode. Formulating EA in a cosmetic base (EA Formula 2) considerably improved the amount deposited onto the skin. However, the formulations contain the uncharged active (pH 4.8), thereby making the technique suboptimal for this purpose.

The results suggest that a DC + PC current profile led to the best delivery of AA for Formula 2 (10% AA). Given the multi-component-character of the formulations and the fact that a considerable fraction of the AA may have also been present in a neutral form; it is unlikely that this effect was entirely due to electromigration, which would normally be the principal transport mechanism for iontophoretic enhancement of AA delivery. It is more probable that the DC + PC current profile increased skin permeability and enhanced passive diffusion of neutral AA through a perturbed SC.

In the case of EA, SPC current profile showed a better EA delivery compared to the DC and DC + PC profiles from simple compositions (pH 4.8). It is probable that electroosmosis, the physical effect of the current itself that may allow an improved passive penetration of the active ingredient, and the organic solvents used in the formula tested, all contribute to the passage of the active ingredient at such a short application time. An improvement of skin deposition at pH 7.0 using a simple solution was observed when the active ingredient was in its ionized form. It will be of interest to formulate a cosmetic composition at neutral pH adequate for iontophoresis, to improve the passage of this phenolic compound through the skin. It is essential to understand the chemical state of the active ingredient in the cosmetic composition in order to select the appropriate current application profile for a good delivery strategy.

To conclude, further robust studies with adapted formulations are needed to improve the efficiency of the technique using different current profiles, with the aim of customizing cosmetic routines towards an improved delivery of an active ingredient.

## 5. Patents

Patent number: EP 3 181 117 A1. Title: Vitamin C composition and an iontophoresis method for its delivery. Date of publication: 21.06.2017. Application number: 15200258.0. Date of filing: 15.12.2015. Applicant: L’Oréal. Inventors: CAZARES DELGADILLO, Jennyfer, PLANARD-LUONG, Thi Hong Lien.

Patent number: EP 3 342 452 A1. Title: Systems including association of ellagic acid and microcurrent. Date of publication: 04.07.2018. Application number: 17305004.8. Date of filing: 03.01.2017. Applicant: L’Oréal. Inventors: CAZARES DELGADILLO, Jennyfer, PLANARD-LUONG, Thi Hong Lien.

## Figures and Tables

**Table 1:** HPLC conditions for the analysis of ascorbic acid (AA) and ellagic acid (EA).

Parameters	AA	EA
System	Dionex Ultimate 3000	Agilent Technologies 1100
Column	Supelco Supelcosil LC-NH2, 5 µm, 250 × 4.6 mm	Kromasil C18, 5 µm, 150 × 4.6 mm
Mobile phase	Acetonitrile 75%/KH_2_PO_4_ 0.05 M (6.8045 g/L) 25%	Methanol 70%/aqueous solution at pH 2.4 30%
Flow rate	2 mL/min	0.8 mL/min
Injection volume	50 µL	20 µL
UV wavelength detection	265 nm	252nm

**Table 2:** Study design (*n* = 6).

Formula	Current Application Profile	Polarity
AA 1 (5% AA; pH 6.7)	DC	negative (−)
AA 1 (5% AA; pH 6.7)	SPC	negative (−)
AA 1 (5% AA; pH 6.7)	DC + PC	negative (−)
AA 1 (5% AA; pH 6.7)	OFF mode (control 1)	-
AA 2 (10% AA; pH 6.0)	DC	negative (−)
AA 2 (10% AA; pH 6.0)	SPC	negative (−)
AA 2 (10% AA; pH 6.0)	DC + PC	negative (−)
AA 2 (10% AA; pH 6.0)	OFF mode (control 2)	-
EA 1 (0.5% EA; pH 4.7)	SPC	positive (+)
EA 1 (0.5% EA; pH 4.7)	DC + PC	positive (+)
EA 1 (0.5% EA; pH 4.7)	OFF mode (control)	-

**Table 3:** Experimental conditions of EA assessment (*n* = 6).

Composition	Current Application Profile	Application Time (min)	Polarity
EA solution pH 4.5	DC	10	positive (+)
EA solution pH 4.5	DC	20	positive (+)
EA solution pH 7.0	DC	10	negative (−)
EA solution pH 7.0	DC	20	negative (−)
EA solution pH 4.5	OFF mode (control)	10	-
EA solution pH 4.5	OFF mode (control)	20	-
EA formula 2	DC	10	positive (+)
EA formula 2	DC	20	positive (+)
EA formula 2	OFF mode (control)	10	-
EA formula 2	OFF mode (control)	20	-

**Table 4:** Deposited amount of EA obtained with EA solution at pH 4.5 and 7.0 and EA Formula 2 applied after 10 min and 20 min current application.

Composition	Deposited Amount (µg/cm^2^)			
	10 min		20 min	
	Mean	SD	Mean	SD
Control solution pH 4.5	0.03	0.03	0.02	0.01
EA solution pH 4.5	0.02	0.00	0.26	0.32
EA solution pH 7.0	0.04	0.01	0.52	0.16
Control EA Formula 2	3.38	2.41	3.65	6.68
EA Formula 2	8.97	4.20	2.15	3.40
